# Activating primary care COPD patients with multi-morbidity (APCOM) pilot project: study protocol

**DOI:** 10.1038/s41533-016-0003-9

**Published:** 2017-02-16

**Authors:** Sameera Ansari, Hassan Hosseinzadeh, Sarah Dennis, Nicholas Zwar

**Affiliations:** 1 School of Public Health and Community Medicine, UNSW Medicine, UNSW Australia, Sydney, NSW 2052 Australia; 2 South Western Sydney Local Health District, Faculty of Health Sciences, The University of Sydney, Sydney, NSW 2141 Australia; 3 Centre for Primary Health Care and Equity, UNSW Australia, Sydney, NSW 2052 Australia; 4 School of Medicine, Faculty of Science, Medicine and Health, University of Wollongong Australia, Wollongong, NSW 2522 Australia

## Background

Chronic obstructive pulmonary disease (COPD), third leading cause of mortality worldwide,^1^ is primarily caused by cigarette smoking in Australia.^2^ COPD often occurs in the presence of multi-morbidity, which is the simultaneous occurrence of two or more chronic conditions; this is a growing concern in a health system focused on single-disease management.^3^ Around 80% of older Australians have multi-morbidity, average prevalence of chronic respiratory disease being 9.5%.^4^

A systematic review of COPD education programs suggests that equipping patients with self-management skills is as important as disease knowledge.^5^ Studies exploring positive effects of chronic disease-management interventions^6^ also underline the lack of studies aimed at empowering patients. Although a recent Cochrane Review^7^ of COPD self-management interventions shows increased health-related quality of life and decreased probability of respiratory-related hospitalisation, similar interventions have not been consistently effective when applied in recent trials.^8–10^ Though interventions aimed at modifying COPD-related health behaviour have great potential, extant literature points to need for self-management programs in primary care.

There is a dearth of interventions involving patients with multi-morbidity, clinicians and other stakeholders, that can be generalisable to other populations.^11^ Patients with multi-morbidity are often excluded from chronic disease-management trials; a Cochrane review^12^ of multi-morbidity interventions found only ten such studies. Our recent qualitative study,^13^ which explored the impact of COPD diagnosis in primary care patients with co-morbidities (co-existing chronic conditions), found suboptimal disease understanding and health-care utilisation. The above evidence demonstrates a need for development and testing interventions to increase self-management capacity in COPD patients with co-morbidities.

Even a brief education session is effective in improving disease knowledge and response to symptoms;^14^ as demonstrated by self-management interventions^10, 15^ on COPD which increased patient self-efficacy. Such programs do not only improve health status and prevent hospital remissions,^16^ but also lead to better patient-provider relationships, self-efficacy and sense of identity.^17^ With no prior study having seemed to focus on improving self-efficacy of COPD patients with co-morbidities in primary care, our study aims to empower these patients by trialling a tailored, practice nurse -delivered self-management program.

## Aims

This pre and post-test pilot study aims to trial the impact and feasibility of a tailored self-management program for primary care COPD patients with multi-morbidity. Our hypothesis is that at six months’ follow-up, participants would have:

1. Better activation in terms of their COPD-related health behaviour.
2. Improved knowledge and self-management capacity of COPD.
3. Increased self-efficacy in terms of their overall health behaviours.

## Methods

This pilot study is funded by GlaxoSmithKline Australia and recognised by the National Health and Medical Research Council through a scholarship to S.A. The study has approval from the Human Research Ethics Committee of UNSW Australia (HREC14139).

### Inclusion/exclusion criteria

General practices will be eligible if they maintain an electronic database of medical records and employ one or more practice nurse (PN).

Patients will be eligible if they are aged between 40 and 84 years, have a recorded spirometry diagnosis of COPD and at least one other co-morbidity. Exclusion criteria are poor understanding of English and/or cognitive impairment.

### Setting

The study will be conducted in general practices across Sydney, Australia. Practices will be recruited from the UNSW Practice Based Research Network that links practices, which have expressed interest and/or have participated in previous research by S.D. and N.Z.

**Fig. 1:**
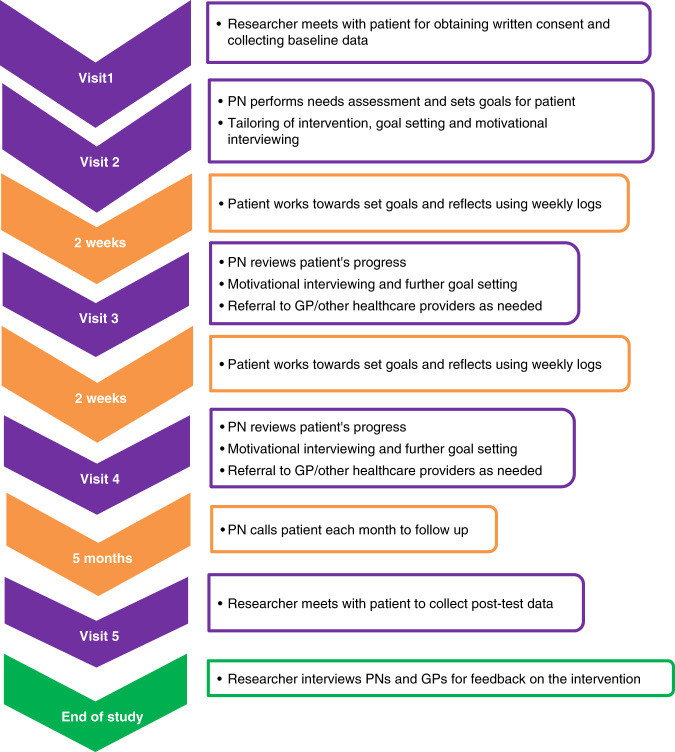
Study protocol

This is a pre and post-test study comprising a mixed method design, with outcome measures to be collected at baseline and six months’ follow-up. The protocol is summarised in Fig. 1.

### Recruitment

An information sheet about the study will be faxed to invite potential practices. S.A. will visit interested practices to answer questions and obtain written informed consent from general physician (GP)s and PNs. Practices who do not respond will be followed up after two weeks.

Staff from participating practices will identify potential patients from their database based on inclusion criteria. PNs will then send an invitation letter to eligible patients and remind non-responders after two weeks by telephone. Interested patients will attend visit 1 at their practice, during which S.A. will answer questions, obtain written consent, and ask them to complete the baseline questionnaires.

Patient recruitment is deemed feasible as chronic respiratory disease accounts for 12% of patient-GP encounters in Australia, COPD being the 11^th^ most common cause for general practice visits.^18^ Recent COPD casefinding studies in Sydney^19, 20^ have found high disease incidence.

### PN training

PNs will attend a one-day work shop, facilitated by the investigators, which will cover basic pathophysiology of COPD, concept of multi-morbidity, the study design and rationale, COPD self-management based on GOLD^21^ (Global strategy for the diagnosis, management and prevention of COPD) and COPD-X^22^ (Australian-New Zealand COPD guidelines), medication adherence, pulmonary rehabilitation, smoking cessation and principles of motivational interviewing. The workshop aims to equip PNs with knowledge and confidence to understand the intervention, and enable them to tailor and deliver it to patients using an assessment and planning template developed by the researchers. PNs’ competency in delivering the intervention will be assessed using case scenarios, with investigators enacting patients and providing feedback. PNs’ understanding of the intervention and feedback on the workshop will be obtained by means of an evaluation form.

### Intervention

The self-management program of this study is based on the Health Belief Model (HBM),^23^ a widely-tested health education theory explaining human decision-making and subsequent behaviour.

The assessment and planning template for the PN-patient sessions incorporates the HBM’s constructs with strategies for self-management of COPD and co-morbidities. The template includes cues to:

1. COPD knowledge^24, 25^
2. Relationship between COPD and patients’ co-morbidities
3. Management and prioritisation of their multiple chronic health conditions
4. Managing flare-ups using a COPD Action Plan^26^
5. Recommended exercises^27^ and pulmonary rehabilitation
6. Proper use of inhalers^28, 29^
7. Importance of flu and pneumonia vaccination^30, 31^
8. Medication review and adherence
9. Advice on overall health and wellbeing
10. Effects of smoking and benefits of quitting, assistance with quitting and abstinence^32^

Links to relevant resources will be uploaded to a website for the participants’ reference.

During the first PN-patient session (visit 2), individual patient needs will be assessed, and the intervention tailored accordingly. In the following two sessions (visits 3 and 4), PNs will use motivational interviewing^33^ to address barriers faced by patients in managing their COPD in the face of co-morbidities, and work towards optimising health behaviour. The patients will maintain a daily log about their health behaviour in between the education sessions. The logistics of intervention delivery is based on the 5A’s approach,^34^ which is an organisational tool devised to help clinicians Ask, Advise, Assess, Assist and Arrange for their patients’ health behaviour.

Cues to action will be provided to patients in the form of motivational fridge magnets and five follow-up phone calls at monthly intervals by their PNs after the last session. During the course of the intervention, GPs will be notified of situations requiring medical attention (e.g., when treatment needs to be intensified) by the PNs.

### Outcome measures

Impact of the program will be determined by change in patients’ level of self-efficacy between baseline (visit 1) and six months’ follow-up (visit 5), using the Patient Activation Measure (PAM) 13,^35^ a highly-reliable, unidimensional scale of knowledge, skills and confidence for self-management of their health.^36^ The PAM classifies patients across four levels of activation, a significant difference being an increase or decrease of at least one level.

Secondary outcome measures include COPD Knowledge Questionnaire (COPD-Q),^37^ COPD Assessment Test (CAT, an indicator of COPD-related quality of life),^38^ Multimorbidity Illness Perceptions Scale (MULTIPleS, to detect change in perceived impact of multi-morbidity),^39^ a COPD-specific version of the MULTIPleS, Morisky Medication Adherence Scale (MMAS-8)^40^ and accuracy of inhaler technique.^41^ Relationship between objectives, hypothesis, outcomes and measures is outlined in Table 1.

**Table 1:** Relationship between objectives, outcomes, measures and hypotheses

Objective	Hypothesis	Outcome	Measure	Visit
To assess the impact of a PN-delivered tailored self-management intervention on COPD patients with multi-morbidity	Tailored self-management education will improve patients’ activation and engagement in their health behaviour, knowledge and self-efficacy of COPD, and increase confidence and skills to manage their multi-morbidity	Change in knowledge, skills and self-management capacity of multiple chronic conditions	Patient Activation Measure (PAM)13^35^	1, 5
		Change in disease knowledge	COPD Knowledge Questionnaire (COPD-Q)^37^	1, 5
		Change in disease related quality of life	COPD Assessment Test (CAT)^38^	1, 5
		Change in perceived impact of multi-morbidity	Multimorbidity Illness Perceptions Scale (MULTIPleS)^39^	1, 5
		Change in perceived impact of COPD in the context of multi-morbidity	COPD-specific version of MULTIPleS	1, 5
		Modified adherence to prescribed medical regimen	Morisky Medication Adherence Scale (MMAS-8)^40^	1, 5
		Accuracy of technique while using inhalers prescribed for COPD	Inhaler device technique checklist^41^	2, 3, 4, 5
To assess the feasibility and sustainability of the PN-delivered self-management education program		Barriers and facilitators to delivery of the intervention, feedback on the program	Semi-structured interviews with GPs and PNs	End of study

COPD chronic obstructive pulmonary disease, PN practice nurse

In accordance with Normalisation Process Theory,^42^ a reliable framework for complex interventions, qualitative feedback will be obtained at the end of the study from participating PNs and GPs to examine feasibility and sustainability of the program.

### Data analysis

Quantitative data will be analysed using Statistical Package for the Social Sciences. Descriptive statistics of responses, expressed as absolute and relative frequencies, will be generated. Categorical data will be analysed by Chi-squared or Fisher’s exact test. Student’s *t*-test for paired samples will be applied for comparing differences in pre and post-intervention outcome measures. Associations will be given as odds ratios.

Qualitative interview transcripts will be coded using NVivo software and thematically analysed by an iterative process.

### Sample size

In accordance with the recommended sample size for feasibility studies demonstrating intervention efficacy in a single group,^43^ our pilot study requires 40 patients, allowing for a 20% drop-out rate^44^ between baseline and six-month visits. This is sufficient to detect a difference in paired measures of patient activation, as demonstrated by a recent study^45^ which used the PAM 13 as an outcome measure, with 85% power at the 5% significance level. A significant difference would be a change between two adjacent stages of the PAM.

### Qualitative process evaluation

Following completion of the program, participating PNs and GPs will be interviewed to gain their feedback. They will be invited via email and written informed consent obtained for recording the interviews, which will be conducted face-to-face by S.A. The interviews will be semi-structured with open ended questions covering their experience and opinions regarding the study, as well as feasibility and sustainability of the program. The interview transcripts will be analysed thematically and coded using NVivo software.

## Discussion

To our knowledge, this study is the first aimed at improving self-efficacy of primary care COPD patients with co-morbidities. Primary care is the ideal setting for implementing self-management interventions because early-stage COPD is typically diagnosed and managed in this setting. Our tailored, PN-delivered education program might not only benefit these patients, but could also upskill the PNs’ role in chronic disease management.

According to the UK Medical Research Council framework for complex interventions,^46^ feasibility studies like ours are a vital step for overcoming problems with acceptability, compliance, intervention delivery, participant recruitment, retention and follow-up, and effect size in larger intervention studies. Our study would be enriched by qualitative feedback from patient participants, but this will extend the data collection process and is hence a limitation.

Experience of intervention delivery and uptake from our pilot study would help refine the intervention and shed light on its acceptability and feasibility in day-to-day general practice, providing an evidence base for upscaling the intervention to be tested in a randomised controlled trial. This capacity-building initiative would also promote collaboration and knowledge translation between the various stakeholders.
